# N-Way FRET Microscopy of Multiple Protein-Protein Interactions in Live Cells

**DOI:** 10.1371/journal.pone.0064760

**Published:** 2013-06-06

**Authors:** Adam D. Hoppe, Brandon L. Scott, Timothy P. Welliver, Samuel W. Straight, Joel A. Swanson

**Affiliations:** 1 Department of Chemistry and Biochemistry, South Dakota State University, Brookings, South Dakota, United States of America; 2 Program in Immunology, University of Michigan Medical School, Ann Arbor, Michigan, United States of America; 3 Center for Live Cell Imaging, University of Michigan Medical School, Ann Arbor, Michigan, United States of America; 4 Department of Microbiology and Immunology, University of Michigan Medical School, Ann Arbor, Michigan, United States of America; University of California, Irvine, United States of America

## Abstract

Fluorescence Resonance Energy Transfer (FRET) microscopy has emerged as a powerful tool to visualize nanoscale protein-protein interactions while capturing their microscale organization and millisecond dynamics. Recently, FRET microscopy was extended to imaging of multiple donor-acceptor pairs, thereby enabling visualization of multiple biochemical events within a single living cell. These methods require numerous equations that must be defined on a case-by-case basis. Here, we present a universal multispectral microscopy method (N-Way FRET) to enable quantitative imaging for any number of interacting and non-interacting FRET pairs. This approach redefines linear unmixing to incorporate the excitation and emission couplings created by FRET, which cannot be accounted for in conventional linear unmixing. Experiments on a three-fluorophore system using blue, yellow and red fluorescent proteins validate the method in living cells. In addition, we propose a simple linear algebra scheme for error propagation from input data to estimate the uncertainty in the computed FRET images. We demonstrate the strength of this approach by monitoring the oligomerization of three FP-tagged HIV Gag proteins whose tight association in the viral capsid is readily observed. Replacement of one FP-Gag molecule with a lipid raft-targeted FP allowed direct observation of Gag oligomerization with no association between FP-Gag and raft-targeted FP. The N-Way FRET method provides a new toolbox for capturing multiple molecular processes with high spatial and temporal resolution in living cells.

## Introduction

Over the last decade, Fluorescence Resonance Energy Transfer (FRET) microscopy became a powerful tool for monitoring intracellular protein associations during signal transduction. Genetically encoded fluorescent protein (FP) fusions and FP-biosensors enabled FRET-based visualization of dynamic signaling events such as imaging the activities of small G-proteins (Ras, Arf, Rho) [1]–[5] to measuring the conformational states of kinesin [6] within living cells. Such experiments rely on drawing comparisons between morphological structures and FRET signals to gain mechanistic insight. However, for FRET microscopy to reach its full potential, simultaneous imaging of multiple molecular events relative to another is needed. FRET microscopy methods using multiple pairs of FP-fusions have been developed for three FP systems [7], [8]. With improvements in the spectral characteristics of FPs, new multifluorophore FRET microscopy methods have the potential to decipher the spatial and temporal interplay of multiple biochemical activities within single living cells.

Achieving multifluorophore FRET measurements requires separation of overlapping spectroscopic parameters. For a given FRET interaction, three spectral components must be resolved: direct donor fluorescence, direct acceptor fluorescence and FRET-induced acceptor fluorescence [9]. Typically however, fluorophores used for FRET have overlapping excitation spectra and emission spectra in addition to donor emission and acceptor excitation overlap. Numerous methods have been devised to correct these additional overlaps for two-fluorophore FRET analysis [10]–[12]. Furthermore, additional calibration methods have been devised to rescale the fluorescence signals and enable measurement of the apparent FRET efficiencies (product of the fraction of donor or acceptor in complex and the fraction of donor energy transferred) and relative concentrations of donors and acceptors [9], [13], [14]. While these methods provide insight into the cellular organization of molecular activities ranging from vesicle transport [3], [15] to regulation of motor proteins [16] and the assembly of HIV virions [17], examination of the interplay between signaling molecules of biochemical pathways has been slowed by lack of robust multifluorophore FRET methods.

Multispectral microscopy platforms have the ability to unmix overlapping fluorescence signals and have been widely used to estimate the relative abundances of multiple fluorophores within a sample [18]–[20]. These ‘linear unmixing’ methods are based on the axiom that the net fluorescence spectrum is defined by the linear superposition of the excitation and emission spectra for each fluorophore in the sample. Recovering the mixture of species in the sample can thus be represented as the ‘linear unmixing’ problem in which the data, contained in vector **d,** are described by the product of the spectral mixing matrix **A** and the abundance of fluorescent species contained in vector **x**,
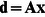
(1)


The mixing matrix includes the spectral signature of each fluorophore on a particular instrument and is sometimes referred to as a spectral library. Linear unmixing recovers the abundance of each fluorophore (**x**) by multiplying both sides of Eq. 1 with the inverse or Moore-Penrose pseudoinverse of **A** (e.g. **A^−1^**) or by using constrained iterative methods [18], [21].

Conventional linear unmixing approaches do not account for FRET processes [22]. This deficiency precludes separation of the three spectral components needed for intermolecular FRET-direct donor fluorescence, direct acceptor fluorescence and FRET-induced acceptor fluorescence and therefore does not allow for estimation of apparent FRET efficiencies or the mole fractions of interacting molecules [9], [14]. It has been shown that the linear mixing model (Eq. 1) can be modified to account for FRET between two fluorophores and this method provides the identical results for the ‘three-cube’ method of FRET Stoichiometry [21]–[23]. In this context, the linear mixing model takes on the following form:
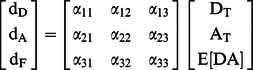
where, the d’s are the images captured under different illumination conditions that are optimal for the donor (D), acceptor (A) or FRET (F), α’s are the FRET spectral components [14], [21], [22] and [A_T_] and [D_T_] are the total acceptor and donor concentrations and E[DA] is the product of the FRET efficiency and concentration of the donor/acceptor complex. This result implies that FRET methods can be formulated in terms of linear algebra, thereby reducing the complexity of multi-fluorophore experiments to allow comparable, quantitative measurements using either filters or spectral detection.

Here, to the best of our knowledge, we provide the first algorithm for generalizing linear unmixing to FRET between any number of fluorophores. This method is similar to that proposed by Woehler [24] for live-cell imaging of multiple FRET-based biosensors. The N-Way FRET methods proposed here accommodate both linked and free fluorophores. We propose and validate calibration methods based on standard FPs expressed in live cells as well as FP-labeled HIV Gag molecules. Finally, we provide a linear algebra-based method for error propagation permitting optimization of FRET experiments.

## Materials and Methods

### Theory

**Figure 1:**
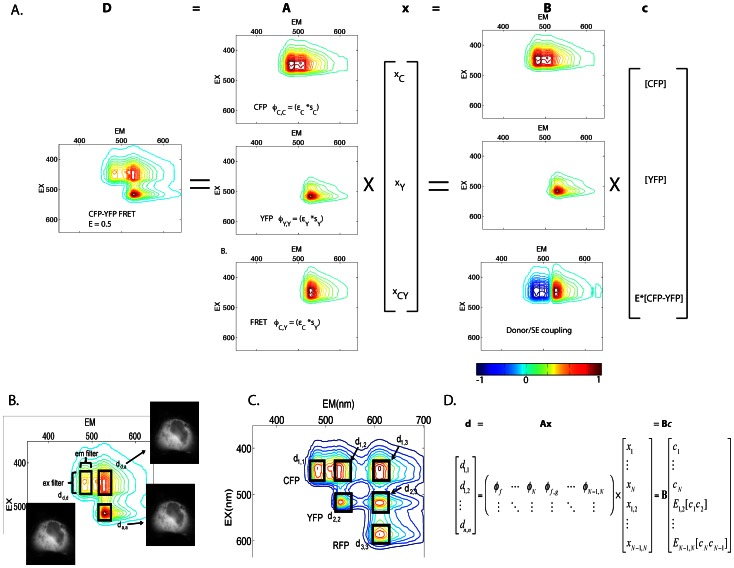
N-Way FRET on the excitation-emission landscape. Spectroscopically, FRET is a coupling between donor excitation and acceptor emission. This excitation-emission coupling (Φ) can be described by the outer product of the excitation vector **ε** and an emission vector **s**. The Φ signatures define the spectral library **A** for the N-Way FRET linear unmixing problem (**d** = **Ax** = **Bc**) that can be viewed on the 2D excitation-emission landscape in addition to viewing the data (**d**). Specifically, these appear as topographical features with light green = 0, warmer colors are increasing height and dark blue colors are negative. **A**) The 2D spectrum for CFP-YFP FRET can be decomposed into the superposition of CFP (Φ_C,C_), YFP (Φ_Y,Y_) and CFP-YFP FRET (Φ_C,Y_). Recovering **ε** and **s** for each fluorophore in the system allows calculation of the unmixing matrix, **A** which can be linearly unmixed to estimate the fluorescence from CFP (x_CFP_), YFP (x_YFP_) and the FRET sensitized emission (x_CY_). **B** can be obtained by calibration with known FRET efficiency standards. Linear unmixing with **B** to allows estimation of concentrations of total fluorophores ([CFP] and [YFP]) and apparent FRET (E_CY_[CFP-YFP]) which are contained in vector **c**. During this step, a negative component (blue color) couples the FRET-associated decrease in donor fluorescence to an increase in acceptor fluorescence. **B**) For most instruments, the complete landscape is not measured, rather, excitation and emission bandpass filters (boxes) define portions of the excitation-emission landscape. For 2-Way FRET the three images needed are d_c,c_, d_y,y_ and d_c,y_. **C**) As more fluorophores are added to the system (e.g. the addition of RFP), the spectral landscape grows by the addition of direct fluorescence components along the diagonal (d_1,1,_ d_2,2_, and d_3,3_) and their possible FRET interactions which appear as off-diagonal peaks (e.g. d_1,2_, d_1,3_ and d_2,3_). **D**) The mathematical form of this problem generalizes to account for multiple fluorophores engaged in FRET.

In this linear unmixing method for FRET microscopy, we use Parallel Factor analysis (PARAFAC) of single fluorophore samples to determine the instrument specific excitation and emission signatures for individual fluorophores. These signatures are then used to compose the possible excitation and emission couplings (EEC) both within (e.g. excitation and emission for one fluorophore) and between fluorophores (e.g their FRET-couplings, excitation of one fluorophore and emission of another, Fig. 1). These EECs are then used to generate a spectral library that when inverted, provides the least-squares estimate of the fluorescence contributions from each fluorophore and each FRET interaction in the system. A second calibration step uses linked FRET constructs to unitize the EECs and couples FRET-associated losses of donor fluorescence to increases in acceptor fluorescence. The result is a linear unmixing model that allows estimation of total fluorophore concentrations and apparent FRET efficiencies (product of the fraction of energy transferred and the fraction of interacting molecules). Extending this linear formalism, we provide a simple method for estimation of uncertainty based on propagation of shot noise into the fluorescence estimates, concentration estimates and apparent FRET efficiency estimates.

During FRET, the donor transfers a portion of its excited state energy to an acceptor. Spectroscopically, this process results in coupling of the donor’s excitation spectrum with the acceptor’s emission spectrum [25]. Therefore, measurement of molecular associations by FRET requires assignment of FRET and non-FRET EECs to interacting (bound) and non-interacting (free) species. An excitation-emission landscape or matrix (a.k.a. EEM) representing the possible EECs of FRET and non-FRET fluorescence can be used to visualize the spectral characteristics of FRET between any number of fluorophores (Fig. 1). For fluorophores unaffected by environmental factors such as pH and ion concentration, the excitation/emission landscape is comprised of the superposition of fluorescence amplitudes that are proportional to the concentrations of those fluorophores. Tri-linear (excitation, emission and concentration) decomposition of the excitation/emission landscape has been used to estimate fluorophore concentrations by PARAFAC [19]. However, PARAFAC-based analysis cannot be used to estimate the concentration of FRET-engaged species since FRET is a linear combination of the excitation spectrum from one molecule and the emission spectrum of another [26]. This limitation can be overcome by modifying the linear unmixing equation using EECs in place of separate excitation and emission spectra. Here, we show that FRET can be described as an EEC which is all-positive and this enables spectral separation of FRET and non-FRET components. Alternatively, we show that FRET can be described as an EEC that contains both positive and negative components for estimating apparent FRET efficiencies. Using this information, we construct a linear mixing model of the form of Eq. 1 capable of accommodating FRET processes by converting these two dimensional EECs into one dimensional spectral contributions to describe the linear superposition of FRET and non-FRET signals.

### Defining the Excitation/Emission Coupling (EEC)

The fluorescence intensity of a particular fluorophore depends on many parameters intrinsic to the fluorophore and the instrument used to detect it. These include the excitation cross section of the fluorophore, the illumination intensity, the transmission properties of the optics, the intersections of bandpass filters and detector sensitivities with fluorophore emission spectra, et cetera [14]. These parameters can be grouped by their contributions to either excitation or emission. Thus, for n excitation wavelengths and m emission wavelengths, the EEC of a particular fluorophore (f) can be given by a trilinear model composed of parallel factors. This model can be stated as the outer product (denoted by ⊗) of an excitation vector (**ε**_f_), emission vector (**s**_f_) and fluorophore concentration vector **x**_f_ which gives rise to a three-dimensional data matrix **D**_f_ [26],
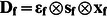
(2)


The excitation vector **ε_f,_** is the integral of the product of the excitation L, during illumination condition i, and excitation spectrum of fluorophore f, ε_f_(λ),
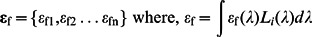
(3)


The emission vector **s**_f_ **,** is the integral of the product of the quantum yield Q_f_ and the emission spectrum s_f_(λ), the j^th^ emission bandpass B_j_ and camera efficiency C(λ) over wavelength λ,
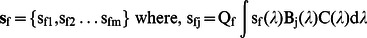
(4)


The EEC is then given by the product **ε**_f_ ⊗ **s**_f_ and the intensity of each fluorophore in each pixel is given in vector **x**_f_ (Fig. 1). For a system containing multiple fluorophores, the trilinear model can be extended to account for the contributions of multiple fluorophores (f = 1, 2, 3, … N) by their superposition,
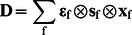
(5)


The vectors **ε**_f_, **s**_f_ and **x**_f_ can be recovered by applying PARAFAC to images or fluorometric data to estimate the spectra and abundances of fluorophore mixtures [19], [26]. The PARAFAC model, however, cannot directly accommodate FRET, since FRET represents a linear combination of **ε** and **s**. Despite this limitation, here, we apply PARAFAC to determine **ε**_f_ and **s**_f_ from cells expressing one fluorophore or mixtures of fluorophores for which no FRET occurs. This information is then used to create an explicit EEC for linear unmixing. Direct application of PARAFAC to the FRET-unmixing problem may also be possible, however it will require modification of the PARAFAC algorithm to account for explicit linear dependencies such as the PARALIND method [27].

### Redefining the Linear Unmixing Matrix to Account for FRET

Once determined, the **ε**_f_ and **s**_f_ vectors can then be used to define the linear unmixing model for FRET (e.g. **d** = **Ax**). The objective is to recover the relative fluorescence from total donors, total acceptors and the FRET-induced acceptor emission. The EEC for direct fluorescence (e.g. light absorbed and emitted by fluorophore f) is the outer product of the excitation and emission:
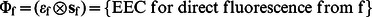
(6)


The EEC for FRET-sensitized emission (fluorescence from fluorophore g resulting from absorption of light by fluorophore f) is the outer product between excitation vector for fluorophore f and the emission vector for fluorophore g:
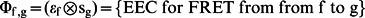
(7)


The amplitude of the EEC is simply given by the abundance of the fluorophores in complex or free. Importantly, this operation is identical to recovering the spectroscopic definition of sensitized emission [21], [22], [25], [28]. In practice, PARAFAC is used to recover the excitation and emission components from measurements made on a specific instrument and fluorophore, rather than relying on published parameters for the absorption and emission spectra, transmission of the optics and extinction coefficients [22]. Thus, the net excitation/emission spectrum (Fig. 1) is given by the superposition of the spectral components for each fluorophore scaled by their abundance and the spectral components of each FRET interaction scaled by the abundance of donor-acceptor complexes:
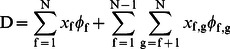
(8)


Here, fluorophores are indexed by energy, starting with f = 1 for the highest energy fluorophore and f = N for the lowest energy fluorophore. This formalism assumes that FRET only occurs from higher energy fluorophores to lower energy fluorophore. Reverse FRET reactions could be accounted for by producing the appropriate x and φ components and adding a third summation to Eq. 8.

To accommodate the linear unmixing problem, Eq. 8 can be arranged into matrix notation, where the matrices **Φ** and data **D** are vectorized (denoted with **φ** and **d**). Here, **φ** comprises the columns of the spectral library **A** (note that f, g are fluorophore indices not the indices of **A**) and **d** would contain the concatenated spectral data (pixels, ROIs, etc.).
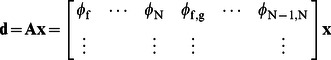
(9)


This solution extends linear unmixing to N number of fluorophores engaged in FRET in any combination. It is important to note that FRET EECs are treated as independent species in **x** and no coupling between loss of donor fluorescence and the sensitized acceptor fluorescence is enforced in this expression.

This model (Eq. 9) will uniquely identify FRET and non-FRET components provided sufficient support in **d.** In particular, solving for **x** requires that the vector **d** contains the same (determined) or more (overdetermined) elements than **x**. This imposes constraints on the experimental data. Specifically, for each fluorophore and each possible FRET interaction in the system, the elements of **d** must be unique (in the linear algebra sense, e.g. they are not linear combinations of the other elements in **d**). This constraint can be satisfied for a multifluorphore system by ensuring that excitation and emission spectra for each fluorophore are sampled in all combinations for excitation wavelength<emission wavelength. In general, the unmixing model will be maximally sensitive when the excitation and emission wavelengths correspond to the EEC peaks (Fig. 1), however optimization of these choices and their bandwidths will require defining and optimizing a cost-function based on a specific noise model [22], [29], [30].

### Unitization of the Unmixing Matrix

In the linear unmixing model above, FRET and non-FRET EECs were treated as separate spectral species; however, these signals are coupled. For example, if the FRET efficiency is 100% and all of the donors are in complex, then the amplitude of the donor signal should be zero. This fact is not enforced in Eq. 9, or in any method that simply unmixes the overlapping fluorescence components. Here we account explicitly for the loss of donor fluorescence and increase in acceptor fluorescence to allow for recovery of FRET Stoichiometry estimates for molar ratio and apparent FRET efficiency for linked and unlinked molecules [14], [31]. As previously shown by Neher and co-workers and our group, quantitative recovery of the total number of acceptors and donors, as well as the apparent FRET efficiency can be obtained from the linear unmixing model ([A_T_], [D_T_] and E[DA]) [21]–[23]. With these terms recovered, their ratios provide the apparent FRET efficiencies E_A_ and E_D_ and the molar ratio R_M_ as previously defined [3], [9], [14], [21]. To define the linear mixing model in a way that returns the concentrations of fluorophores and apparent concentrations of complexes (e.g. E[DA]) we seek a new matrix **B** such that.
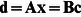
(10)where **c** contains the concentrations of donors, acceptors and the apparent FRET complex in the same units.

This modification requires that the contributions of fluorescence loss from donors and corresponding emission enhancement from acceptors be accounted for explicitly in **B**. This requires that all of the terms in **c** have the same units thereby allowing the relative contributions of various species to be coupled. We propose a matrix with the scalar unit conversions **γ** along its diagonal (**Γ** = diag(**γ**)) to unitize the columns of **A.** Next, we define the donor/acceptor interactions via an interaction matrix, **M**, populated with signed binary (e.g. 0, 1, −1). With this definition, we can write **B** as,
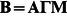
(11)


Specifically, **M** has 1 along its diagonal (each fluorophore f and each FRET pairing f,g are represented) and −1 in each row corresponding to all positions where there are possible FRET interactions (e.g. where the fluorophore index g is non-zero). In the case of 3-Way FRET for a determined system (e.g. **d** and **c** are of equal length), with the fluorophores CFP (C), YFP (Y) and mCherry (R), **M** would be a 6×6 matrix with 1 on the diagonal and −1 for each possible FRET coupling e.g.
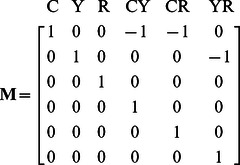



Thus, for a linked construct in which each fluorophore is at equimolar concentration and the FRET efficiency is known, the unit conversions γ can be found by.
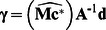
(12)


Where the ‘hat’ indicates the Hadamard inverse. Here, c* contains the ‘known’ concentrations of each fluorophore and the corresponding apparent FRET efficiencies.

Equation 12 suggests a simple calibration, independent of external information or adjustable parameters, but only requiring the measurement of **d** given **M**, c* and **A**. In the case where a single measurement can be made on one construct of known FRET efficiencies, **γ** can be found by simply setting c* to the appropriate concentrations of fluorophores and FRET efficiencies. For example, in the case of a triple fusion of CFP-YFP-RFP, the known concentration vector of arbitrary units would be c* = [1 1 1 E_CY_ E_CR_ E_YR_]. Alternatively, if a single construct containing all fluorophores with known FRET efficiencies cannot be constructed, **γ** can be determined from multiple donor acceptor pairs with known FRET efficiencies. In this case, each donor-acceptor pair would be measured separately to give a set of **γ** vectors: e.g. **γ**_1,2_, **γ**_1,3_, **γ**_2,3…_ **γ**_f,g_. Each of these vectors contains an element that must have the same units as another member of the set. For example, pairwise linked constructs of CFP, YFP and RFP would have **γ**_C,Y_, **γ**_C,R_, **γ**_Y,R_. In this set, the units of [YFP] would appear in both **γ**_C,Y_ and **γ**_Y,R_. By making the overlapping elements equal (e.g. renormalizing the pairwise set of **γ** vectors) to [YFP] and then repeating the process for [RFP] a single common **γ** can be determined.

### General Procedure for Obtaining and Solving the N-Way FRET Equation

The above theory defines the procedure for quantifying FRET interactions between N-fluorophores. This approach should be generally applicable to any instrument capable of recording excitation and emission combinations centered on each fluorophore in the system.

The steps for calibration of this method are:

1. Collect reference excitation and emission spectral signatures for each fluorophore. This can be accomplished by expressing each fluorophore separately or in mixtures where no FRET occurs. Data must be captured using filter combinations for each FRET and non-FRET EEC in the system.
2. Use PARAFAC to determine **ε**_f_ and **s**_f_ for each fluorophore.
3. Construct all possible FRET and non-FRET EECs (e.g. **φ**_f_ and **φ**_f,g_).
4. Construct the unmixing matrix **A**. (Stop here if relative FRET and non-FRET fluorescence measurement are sufficient).
5. Measure linked constructs with known FRET efficiencies to determine **γ** and generate **B**.

Once the unmixing matrix **A** or the unitized unmixing matrix **B** have been determined, the abundances and apparent FRET efficiencies can be determined by directly solving the linear system of equations. For a determined case, **d** has the same number of elements as **x** or **c**, and the system can be inverted to find the least squares solution [22], [32], [33].
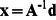
(13)

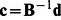
(14)


Alternatively, for overdetermined systems, such as would be encountered with hyperspectral detection, the system of equations can be solved by using the Moore-Penrose pseudoinverse of **A** or **B.** These solutions provide the ideal estimate of **x** or **c** in the case where the noise corrupting the data is Gaussian [18], [21]. Alternative methods such as Maximum Likelihood could be devised for solving these equations for other noise model cases including the more relevant Poisson noise model on **d** [21].

### Estimation of Uncertainty

Formulation of FRET as a linear unmixing problem provides a simple method for the propagation of uncertainty in the data into the estimated fluorescence amplitudes and the concentrations. If the uncertainty in **d** is given by the variance-covariance matrix (**Σ** ^d^) then the uncertainty can be propagated onto **x** or **c** using the general linear error propagation [32],
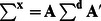
(15)


or
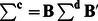
(16)where the variance-covariance matrix is given by:



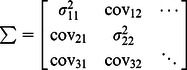
(17)Here, the indices refer to the elements of **d** (e.g. σ_11_^2^ an image of the variance for the first image in **d** and cov_12_ the covariance for the first and second images in **d** etc.).

Thus, we must now define the variance and covariance in our system. In the simplest scenario, most of the uncertainty in the data derives from photon shot noise. Here, the number of photons arriving at a detector obey Poisson statistics and the covariance terms in Σ^d^ are zero. Thus, the variance terms along the diagonal are given by the number of photons in each pixel. For a CCD camera (when operating above read noise), the relationship between camera units versus the variance provides the conversion between CCD units and photons [34]. Other sources of noise including read noise and lamp flicker could be modeled as well. For shot noise only on a CCD camera, the covariance terms are zero and the diagonal of **Σ** ^d^ is given by,
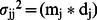
(18)where, m_j_ is the photon conversion for the detector measuring emission j and d_j_ is the j^th^ image of **d**. With this definition, the uncertainty arising from shot noise can be propagated into the estimates for fluorescence and concentration using Eqs. 15 and 16. The coefficient of variation, CV = σ/μ, where μ is the mean in the corresponding image provides a simple way to view the fractional uncertainty [23] and is used here. We anticipate that this formalism will enable testing of error-reduction approaches and using Monte-Carlo simulation and Bayesian analysis [29], [30].

### Definition of Apparent FRET Efficiencies and Molar Ratios

Interpretation of FRET data for intermolecular interactions is simplified by computing ratios of concentrations of total fluorophores and apparent complex concentrations [9]. In the formalism of FRET Stoichiometry, these ratios range from 0–1 and depend on the fraction of donor or acceptor in complex, times the FRET efficiency [9], [14]. In the unmixing method presented here, the apparent FRET efficiencies (E_A_ and E_D_) and the molar ratios (R_M_) of one fluorophore to another can be computed from ratios of the terms contained in the concentration vector **c**. For example, in the case of intermolecular FRET with CFP, YFP and RFP, **c** = {[C], [Y], [R], E_CY_[CY], E_CR_[CR], E_YR_[YR]}. We can write the FRET Stoichiometry ratios for a particular pairing (e.g. CFP and YFP) as,
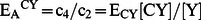
(19)

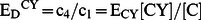
(20)

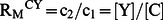
(21)


Additionally, the errors contained in **Σ** ^c^ can be propagated directly into these terms by standard nonlinear propagation [32]. For a ratio of any terms in **c** with associated error matrix **Σ** ^c^, the standard deviation can be found as:
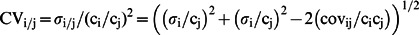
(22)


### Cells and Transfection

COS7 cells were obtained from the American Type Culture Collection (Manassas, VA) and maintained at 37°C under 5% CO_2_ in Dulbecco’s Modified Eagle Medium (HyClone, supplemented with 10% heat-inactivated FBS, 100 U/mL penicillin and 100 µg/mL streptomycin). Cells were plated at ∼3×10^5^ cells onto 25 mm round No. 1.5 coverslips (Fisherbrand, ThermoFisher Scientific, Fairlawn, NJ) and transfected with either FuGENE6 according to the manufacturer’s protocol (Roche, Basel, Switzerland) or jetPEI transfection reagent (Polyplus Transfection, Strasbourg, France) according to the manufacturer’s recommendations. Media was replaced 16 hours after transfection and cells were imaged 24 hours after transfection.

### Constructs

Fluorescent proteins used in this study included: the cyan fluorescent protein (CFP) mCerulean [35], the yellow fluorescent protein(YFP) mCitrine [36], a red fluorescent protein (RFP) mCherry [37] and the non-fluorescent protein YFPdark mCitrineY67C [38], all with “monomeric” A206K mutations [39]. FRET-positive linked fluorescent protein constructs were generated as described in Methods S1 and summarized in Table S1. A low FRET efficiency construct was created by cloning Kif1a in between CFP and RFP [6]. Plasmids containing HIV-Gag and the Fyn(10) signal sequence were gifts from Dr. Akira Ono (University of Michigan, Ann Arbor, MI) and were modified as described in Methods S1 to create CFP, YFP and RFP fusions.

### Imaging

Two microscopes were used in this study. The first (Scope 1) was described previously [21]. Briefly, this instrument was a custom-built Nikon TE2000, which was modified to accommodate two emCCD cameras, emission filter wheels and a rapidly switchable light source. The second instrument (Scope 2) was a custom-built iMIC (Till Photonics USA, Rochester, NY) and is described in detail in Methods S1 (Fig. S1). Both systems utilized multiple cameras for detection, however, Scope 2 had three emCCD cameras (2– Andor iXon 885 and 1 - Andor iXonX3 885), allowing capture of fluorescence data from three fluorophores simultaneously, thereby providing advantages in speed and photon efficiency over Scope 1.

### FRET Efficiency Measurements by Fluorescence Lifetime and Acceptor Photobleaching

The FRET efficiency of linked calibration standards were determined by fluorescence lifetime of the donor using time-correlated single photon counting of cells illuminated by a picosecond Ti-Sapphire Laser, as previously described [9], and using fluorescence bandpass filters to selectively capture the CFP or YFP emission (Fig. S6). Determination of the FRET efficiency for the triple-linked construct was achieved on Scope 2 by sequentially and selectively photobleaching RFP and YFP, using 561 nm or 515 nm lasers, respectively (Till Photonics) (Fig. S5). Additionally, dual construct FRET efficiencies were measured by acceptor photobleaching (Fig. S6) and were found to give comparable results. Both lasers produced collimated illumination fields in the sample plane via a lens that focused them onto the back focal plane. FRET efficiency was computed as the increase in CFP and YFP fluorescence following bleaching of RFP and the increase in CFP fluorescence following bleaching of YFP. Control bleaching experiments demonstrated that no incidental bleaching of the donors occurred during the experiment.

### Image Analysis and Computation

All calculations were performed in Matlab (versions 7.3 and 2009a, Mathworks, Natick, MA) in conjunction with the DipImage toolbox (http://www.diplib.org/, Quantitative Imaging Group, Delft University of Technology, Netherlands) and the N-Way Toolbox for PARAFAC analysis http://www.models.life.ku.dk/ [40]. A graphic user interface for 3-way N-Way FRET analysis has been developed and added to the FRET calculator, which can be obtained from the Center for Live Cell Imaging at the University of Michigan (http://sitemaker.umich.edu/4dimagingcenter/center_for_live-cell_imaging_home).

### Preprocessing

All data images were preprocessed by subtracting camera bias and shade-correcting the images as previously described [14], [21]. Briefly, images for each camera were captured while blocking all light to obtain the bias level. Residual background was subtracted from cell-free regions if present (generally less than 5% of the cellular signal). Images of a thin solution of a fluorescent protein mixture were used to correct the illumination pattern across the field of view for each excitation [14]. Images from the two cameras (Scope 1) or three cameras (Scope 2) were aligned using a projective/affine transform as previously described [21].

### Calibration

To generate **A,** images were captured of cells expressing CFP, YFP and RFP separately. These images were preprocessed and assembled into three image vectors **d**_C_, **d**_Y_ and **d**_R_. Regions of interest (ROI) were drawn over cells to measure their intensities to create trilinear data matrices **D**_C_, **D**_Y_ and **D**_R_. These data were decomposed by PARAFAC analysis according to Eq. 5 to produce **ε**_f_ and **s**_f_ vectors. The EECs were then computed and matrix **A** was assembled as in Eq. 9. To obtain the unitized unmixing matrix, images of cells expressing dual or triple linked constructs with known FRET efficiencies were expressed in cells and images were captured to generate **d** and ROI were drawn to obtain the cellular intensities. After determining **Γ**, **B** was obtained by applying Eq. 11.

### Analysis

Images of cells were captured on the microscope with the same settings as used for calibration, with the exception that exposure could be varied by the user and corrected by rescaling the images after preprocessing by the ratio of the exposures. Following preprocessing, the data were unmixed by applying Eq. 13 or 14. Error analysis was performed as described in the theory section, using the preprocessed data to generate the variances along the diagonal of the variance-covariance matrix.

### Statistical Analysis

For key pieces of data, statistical significance was judged by 1-way ANOVA followed by Tukey HSD post hoc comparison of means using JMP (SAS Institute, Cary, NC).

## Results

### Calibration and Unmixing

To measure matrix **A**, a calibration dataset consisting was measured from regions of interest taken from living cells separately expressing CFP, YFP or RFP (∼20 cells per condition) using excitation and emission combinations for **d** = {cc, cy, cr, yy, yr, rr}, (lower case pairings denote excitation-emission combinations e.g. cr is CFP excitation and RFP emission, Fig. S1). The resulting trilinear data structure was decomposed by PARAFAC to recover FRET and non-FRET EECs, Φ_f_ and Φ_f,g_, which in turn provided **A** per Eq. 9.
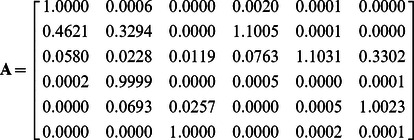



The columns of **A** reflect the non-FRET fluorescence components (columns 1–3) and the FRET fluorescence components (columns 4–6) for **x** = {x_C_, x_Y_, x_R_, x_CY_, x_CR_, x_YR_}. As expected, peak values in each column correspond to the data vector element with the highest selectivity for that species (e.g. **d** = {cc, cy, cr, yy, yr, rr}).

**Figure 2:**
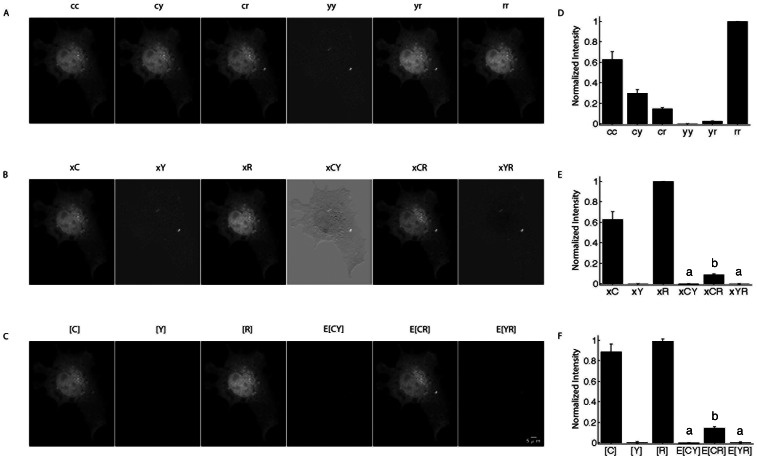
Linear unmixing of FRET amplitudes and N-Way FRET analysis in living cells. **A**) Images of live COS7 cells expressing a CFP-RFP linked construct were captured using paired excitation and emission filter combinations (e.g. cc = CFP excitation, CFP emission) to sample the N-Way FRET landscape. **D**) ROIs to provide the raw intensities from these cells. Unmixing of these images using matrix **A** recovers the fluorescence abundances (e.g. xC) and showed that FRET could be observed only between CFP and RFP (e.g. xCR) (**B,** images are scaled independently) and was reproducible over multiple cells (**E**). **C**) Quantitative unmixing with N-Way FRET showed that equal abundances of CFP and RFP were present and FRET was observed as E[CR], but no was observed for E[CY] or E[YR] as expected (**F**). 20 cells per condition; data from Scope 2; bars not connected by the same letter are significantly different (p<0.05) by Tukey HSD post hoc comparison of means; error bars are standard deviation.

The first test was to determine if **A** could unmix image data from cells expressing linked and unlinked FRET pairs. Indeed, linear unmixing with **A** as in Eq. 13, recovered the correct FRET and non-FRET signals (Fig. 2).

To test the unitized unmixing method, the unitized unmixing matrix **B** was obtained by two approaches. First, the FRET efficiencies were determined from a triple construct of CFP-YFP-RFP by sequentially photobleaching the acceptors. This returned FRET efficiencies of E_CY_ = 0.25+/−0.01, E_CR_ = 0.11+/−0.01 and E_YR_ = 0.18+/−0.01 (Fig. S5). These values were then used to compute **γ** and compose the unitized unmixing matrix **B** per Eq. 11.
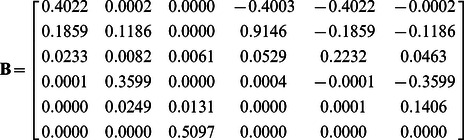



**Figure 3:**
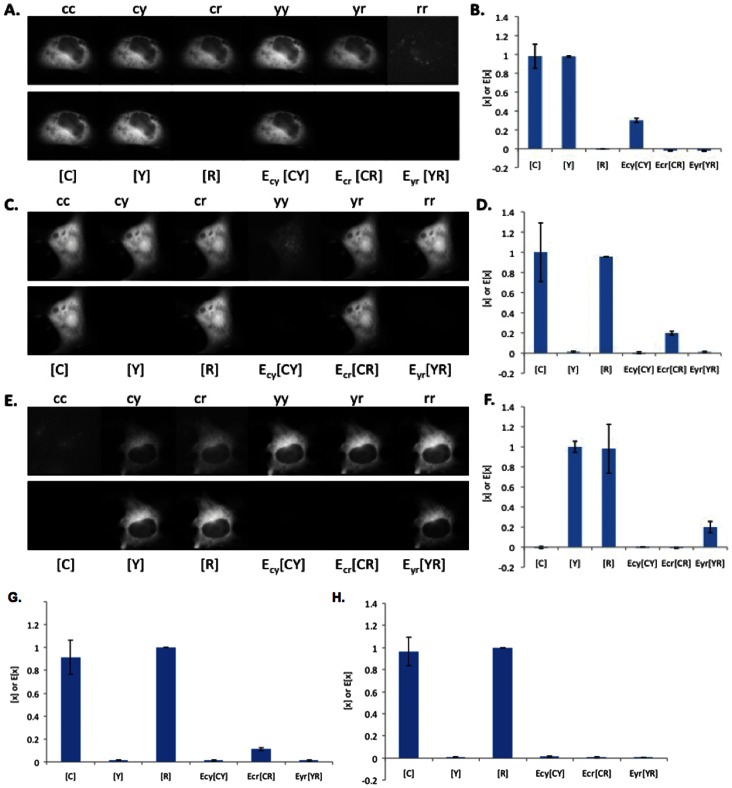
N-Way FRET recovers concentrations and apparent FRET efficiencies from cells expressing FP-FP fusions. Cells were transfected with CFP-YFP (**A** and **B**), RFP-CFP (**C** and **D**), RFP-YFP (**E** and **F**), RFP-darkFP-CFP (**G**) and RFP-kinesin-CFP (**H**). The raw images (top rows, independently scaled) were analyzed by N-Way FRET, using **B^−1^**, to produce the concentration estimates (bottom rows) ([FP], display scale, 0–1,400 intensity units) and the apparent FRET efficiencies (E_FP-FP_[FP-FP], display scale 0–500 intensity units). Images are representative of 20 cells per condition. Plots of concentration estimates (**C, D**, **F**, **G** and **H**) indicated that N-Way FRET accurately recovered the correct one-to-one stoichiometry of each FP in the sample as well as their apparent efficiencies (n = 20 for each). Note the decreasing FRET efficiency observed for increasing size of inserted peptide or proteins: high FRET (RFP-CFP, **D**), low FRET (darkFP, **G**) or no FRET (kinesin, **H**) seen in E_CR_[CR]. Data from Scope 1.

As expected, **B** contains negative values corresponding to the subtractive components for FRET-induced donor losses as suggested by the negative topology expected in the 2D spectrum (Fig. 1). Similar results were obtained for **B**, when pairs of linked FRET constructs were used. The FRET efficiencies of tandem linked constructs CFP-YFP, CFP-RFP and RFP-YFP were determined by both fluorescence lifetime and acceptor photobleaching (Fig. S6). Here, **B** was then computed by finding the common **γ** values (see theory). Both methods for determining **B** produced similar results (compare Fig. 3, Scope 1, with all other figures). Furthermore, good agreement was observed for FRET efficiency determination by acceptor photobleaching and by fluorescence lifetime (Fig. S6), indicating that either method can be used equivalently for calibration of test constructs for N-Way FRET.

We tested N-Way FRET analysis by using the unitized matrix **B** to compute estimates for concentrations of total and FRET complexes (**c** = {[C], [Y], [R], E_CY_[CY], E_CR_[CR], E_YR_[YR]}). As expected, linear unmixing per Eq. 14 with **B** was able to return the correct FRET efficiencies and fluorophore abundances for the tandem constructs (Fig. 2). Unmixing with **B** has a distinct advantage over unmixing with **A** in that it reduces the ambiguity in distinguishing FRET signals from small fluctuations near zero (Fig. 2) and contaminating fluorescence signals from transfection reagents (examples in Fig. S2). For the remainder of the paper, N-Way FRET will refer to unmixing with the unitized matrix **B**.

### N-Way FRET Enables Quantification of Fluorophore Abundance and Apparent FRET Efficiencies

N-Way FRET was tested for its ability to recover the abundances and apparent FRET efficiencies of single FP, FP-FP fusions and FP-FP-FP fusions alone and in the presence of free (non-FRET) FP. N-Way FRET could accurately determine the presence of single FPs expressed within living cells (Fig. S2) indicating N-Way FRET could identify free FPs accurately. In cells expressing FP-FP constructs of known FRET efficiencies (E_CY_ = 0.25, E_CR_ = 0.11 and E_YR_ = 0.18), N-Way FRET accurately recovered the relative concentrations and FRET efficiencies indicating that it could quantify FRET efficiency accurately (Fig. 2). Additionally, N-Way FRET correctly determined the abundances and FRET efficiencies when free, non-interacting fluorophores were present with an FP-FP FRET pair demonstrating that it could deal with mixtures of the two (Fig. S3).

To validate the sensitivity of N-Way FRET to changes in the FRET efficiency, we increased the spacing between the FPs. In addition to the FP-FP constructs above, double fusions were constructed with FPs separated by a nonfluorescent FP carrying the “Amber” mutation, Tyr67Cys [38] (e.g., R-darkFP-C) or a kinesin heavy chain (eg. R-kinesin-C) [6]. The spacing between the FPs should increase from approximately: ∼2 nm for the 27 aa linker (random flight model) to ∼6 nm for the darkFP insert (counting linkers) and greater than ∼60 nm for the kinesin. For these constructs, N-Way FRET showed decreasing FRET efficiency as seen by the relative drop in E_CR_[CR] (Fig. 3G,H); consistent with FRET efficiencies previously observed in orthologous constructs [6], [38]. Together, these data demonstrate that N-Way FRET is able to quantitatively recover the concentrations of interacting FPs and their FRET efficiencies when expressed in pairs and that C, Y and R are adequate donors and acceptors for this method.

**Figure 4:**
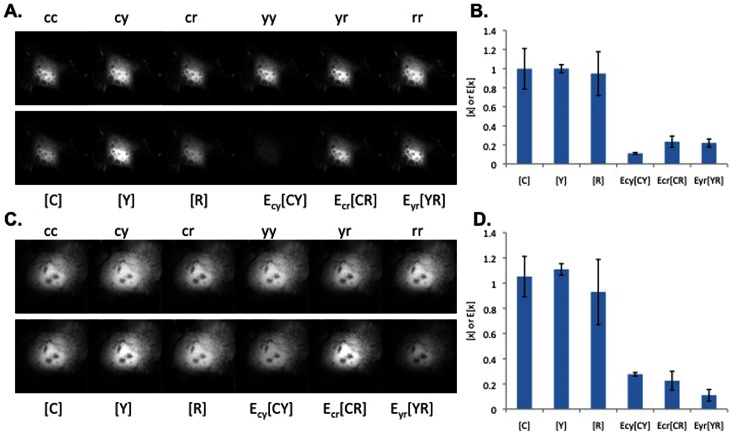
N-Way FRET recovers the relative concentrations and apparent FRET efficiencies from cells expressing FP-FP-FP fusions. Cells were transfected with CFP-RFP-YFP (**A** and **B**) and YFP-CFP-RFP (**C** and **D**) linked constructs. Spectral FRET data (top row) were unmixed by N-Way FRET to produce the concentration estimates (bottom rows) of the total FP (bound and free, [FP]) and the apparent complex concentrations (E_FP-FP_[FP-FP]). Plots of concentration estimates (**C** and **D**) averaged over 20 cells indicate that N-Way FRET recovered correct one-to-one stoichiometry of all three FPs in the sample and that the FPs on the ends of the polypeptide gave the lowest FRET efficiencies (error bars are standard deviation, 20 cells for each, from Scope 2).

To examine the ability of N-Way FRET to recover concentrations and efficiencies from samples containing three fluorophores, we generated FP FRET trio constructs (C-R-Y, C-Y-R). While the amount of FRET in these cells is not apparent in the raw data, N-Way FRET analysis revealed FRET between all FPs and returned the expected equimolar concentrations of each FP and FRET signals that scaled with the spacing between FPs (Fig. 4). Cells expressing C-R-Y demonstrated the highest FRET efficiency between C-R, and Y-R, and lower FRET efficiency for C-Y, consistent with the larger distance between C and Y (Fig. 4). In the C-Y-R construct, the lowest FRET efficiency was between the terminal FPs (C and R), as expected (Fig. 4). While the potential for energy migration from C->Y->R exists, N-Way FRET (or any other spectral method) cannot distinguish this from direct transfer C->R. The low FRET efficiency observed for the terminal FPs in both the C-R-Y and C-Y-R constructs suggests energy migration does not contribute substantially. This observation was supported by C-darkFP-R having nearly the same value for E[CR] as in C-Y-R indicating that Y was not significantly facilitating transfer of energy from C to R (Fig. 3 and 4). Together, these data indicate that N-Way FRET can accurately recover the abundances and apparent FRET efficiencies for model constructs.

### N-Way FRET Enables Imaging of Three-color Intermolecular Interactions in HIV

**Figure 5:**
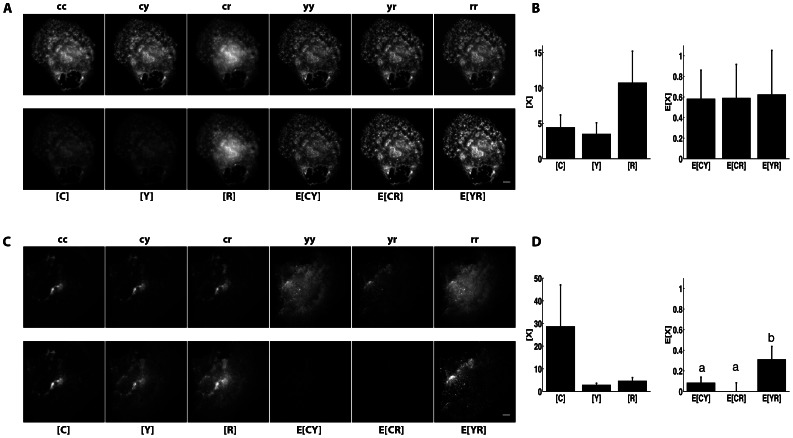
N-Way FRET measures HIV-Gag oligomerization but no association with lipid rafts in a single cell. **A**) COS7 cells transfected with CFP-Gag, YFP-Gag and Cherry-Gag and imaged by N-Way FRET display strong FRET signals on small punctate structures (display ranges: [FP] = 0–30, E[FP FP] = 0–3 intensity units). **B**) Quantification of these signals confirmed that Gag oligomerization resulted in close apposition of the three fluorophores that permitted detection of FRET (n = 8 cells). **C**) Cells transfected with YFP-Gag, Cherry-Gag and the lipid raft marker CFP-Fyn(10) only showed FRET between YFP-Gag and Cherry-Gag (display ranges: [C] = 0–150, [Y] & [R] = 0–20, E[FP FP] = 0–6). **D**) Quantification of N-Way FRET results from C) indicate minimal FRET with CFP-Fyn, but high FRET for Cherry-Gag and YFP-Gag (n = 3 cells, Scope 2, bars not connected by the same letter are significantly different (p<0.05) by Tukey HSD post hoc comparison of means; error bars are standard deviation).

We tested the ability of N-Way FRET to measure intermolecular interactions. Previously, we showed that HIV Gag self-assembly gave high FRET efficiency when expressed as C-Gag and Y-Gag fusions but not when Gag was expressed with a lipid raft-targeted construct Fyn(10)-CFP (which is myristolated and palmitoylated) [17]. To assess N-Way FRET in the context of a macromolecular complex, we co-expressed C-Gag, Y-Gag, and R-Gag. These molecules assembled into virus-like particles that displayed high levels of FRET between all three FP fusions (Fig. 5A,B). As expected from [17], when C-Gag was replaced with Fyn(10)-CFP, minimal FRET was observed between Fyn(10)-CFP and either Y-Gag or R-Gag, despite high levels of FRET persisting between Y-Gag and R-Gag (Fig. 5C,D). These results demonstrate that N-Way FRET can quantify three-way molecular interactions.

### Error Propagation and FRET Stoichiometry in N-Way FRET

**Figure 6:**
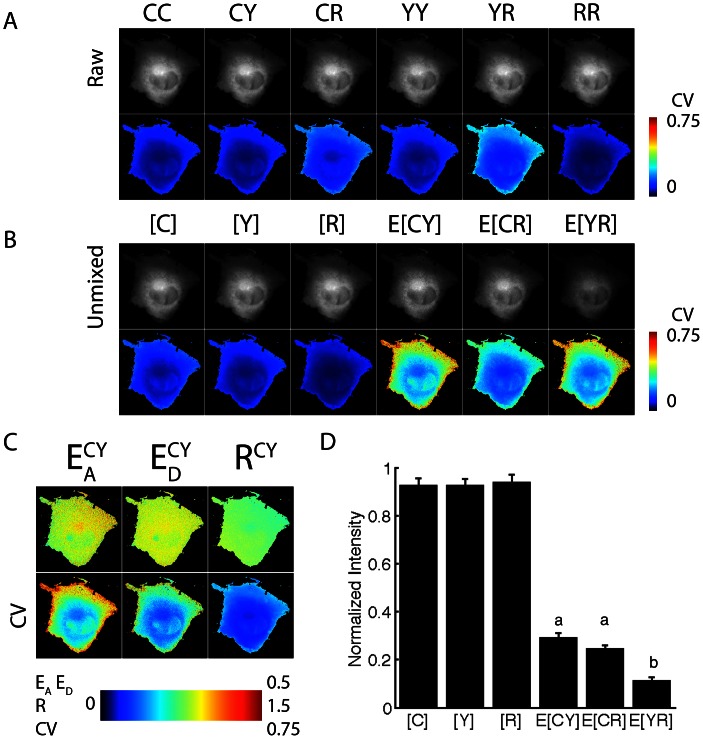
N-Way FRET error propagation for YFP-CFP-RFP linked construct. **A**) The coefficient of variation (CV) was computed from the raw images of a cell expressing the YFP-CFP-RFP linked construct using the photon conversion factor for the CCD cameras. **B**) Unmixed measurement of the total fluorophores and apparent complex concentrations were estimated, along with their error-propagated CV. As expected, the weakest FRET signals showed the greatest CV (warm colors on the periphery of the cell). **C**) Stoichiometric FRET ratios for the CFP-YFP FRET (CR and YR shown in Fig. S4) demonstrated uniform apparent FRET efficiencies (E_A_ and E_D_) and molar ratios (R). The CV (bottom row) indicates that the error was greatest in the periphery of the cell in the E_A_ image. **D**) Quantification of the concentrations from 10 cells. Error bars are standard deviation; data from Scope 2, bars not connected by the same letter are significantly different (p<0.05) by Tukey HSD post hoc comparison of means.

Demonstration of error propagation is shown for cells expressing the Y-C-R FRET trio construct (Fig. 6). The noise per pixel in the input data **d** was determined as the using equation Eq. 18 to create Σ^d^. Unmixing and propagating the error per Eq. 16, permitted determination of the concentrations of FRET and non-FRET signals with associated error matrix Σ^B^. Displaying the CV from Σ^d^ and Σ^B^ illustrated that the cellular periphery had the largest detection and propagated errors as would be expected for the lower signal in this dimmer region of the cell. Additionally, higher propagated uncertainty was associated with signals for which the unmixing operation employed the most additions and subtractions (Fig. 6). This can be seen by noting that the average intensities for E[CY] and E[CR] are approximately equal, however, their propagated CVs differ by about a factor of two (Fig. 6). Thus, the propagated error is a mixture of the initial signal strengths and the weightings of the unmixing operations.

In addition to accurately unmixing 3-Way FRET signals, N-Way FRET can reproduce the apparent FRET efficiencies and molar ratios of FRET Stoichiometry [3], [9]. Furthermore, N-Way FRET defines the error propagation for these images. By computing ratios of the images contained in **c**, per Eqs. 19–21 and propagating the error per Eq. 22, the apparent FRET efficiencies for E_A_ E_D_ and R_M_ were computed from the 3-Way FRET data (Fig. 6 and S4). These results showed uniform FRET efficiencies throughout the cytosol of the cell. Increased variation in the FRET Stoichiometry parameters near the edges of the cell are well predicted by the increase in CV in these regions.

## Discussion

Here we developed and tested a simple formalism to generalize spectral FRET analysis to any number of interacting FRET pairs using instrument-derived calibrations. This linear algebraic formalism eliminates the need for analytical expressions and explicit assumptions about fluorophore and instrument parameters. In doing so, N-Way FRET should be generally applicable to any microscope, fluorometer or cytometer in which spectral detection can be made using combinations of excitation and emissions. In addition, this method opens up the possibility of characterizing and removing additional unwanted fluorescence components, such as autofluorescence [19], [33]. For example, autofluorescence could be added as a spectral component to either the **A** or **B** spectral libraries by application of PARAFAC to cells that have only autofluorescence, provided at least one additional excitation-emission combination was available beyond what was required by the FRET pairs. Lastly, this method provides convenient error propagation directly from raw data into the estimates of total and interacting fluorescent molecules. We demonstrate the utility of this method for idealized linked constructs and interactions between multiple fluorescently-labeled molecules during HIV assembly.

At the heart of this technique is the redefinition of the linear unmixing problem in terms of an excitation-emission spectral landscape (Fig. 1). Each signature on that landscape defines a column in the unmixing matrix. By defining the FRET and non-FRET components as the outer product of excitation and emission vectors, the linear algebraic method of PARAFAC can be used to obtain these excitation and emission components from measurements made on the microscope. Since the calibration is performed using data collected from free, cytosolic fluorophores, this method eliminates any need for external assumptions about fluorophore characteristics, spectral throughput of the instrument, quantum efficiency of the cameras or other instrument-specific parameters. In some cases it may be difficult to recover fluorescence data that are devoid of contaminating signals for the calibration. We expect that principle component analysis or non-negative matrix factorization should allow for refinement of calibration data prior to PARAFAC to identify and eliminate these contaminating signals [41]. The non-unitized unmixing resulting from these efforts enables measurement of FRET and non-FRET fluorescence signatures while defining the propagation of uncertainty via matrices **A** and **Σ** ^d^. By applying a second set of linked FRET standards with known FRET efficiencies, a unitized unmixing matrix, **B,** allows estimation of concentrations (**c**) of total and apparent complexes with error matrix **Σ** ^c^. Ratios of components within **c** provide all of the terms defined by the FRET Stoichiometry method (molar ratios, apparent donor efficiency, apparent acceptor efficiency) thereby capturing all of the information available from a spectral FRET measurement with the added capability of defining these terms for an arbitrary number of fluorophores.

This work was motivated by the demonstration by Neher and co-workers that detection of FRET could be cast as a linear unmixing problem [22], [23] and previously, we demonstrated that the analytical expressions of FRET Stoichiometry could be restated as a linear unmixing operation [21]. Woehler demonstrated that using published spectral data and additional reference measurements, that lux-FRET can be adapted similarly to N-Way FRET for three fluorophores and enables measurement of three biosensors simultaneously [24]. These recent works are converging on the idea that linear algebraic forms are needed to enable new FRET microscopy methods. Previously, a number of papers presented 3-way FRET methods using analytical expressions [7], [8]. However, in each of these cases, explicit assumptions and extensive prior information about fluorophore and instrument parameters are required. Such assumptions limit the applicability of these methods to particular instruments and fluorophore combinations. Furthermore, the added complexity of multiple fluorophores competing for spectral bandwidth necessitates a quantitative treatment of error propagation, which, thus far has been completely lacking. The N-Way FRET method overcomes these limitations, allowing for a more diverse range of fluorophores, instrument usage and error analysis. Examples of future applications for N-Way FRET not accessible to any current methods include imaging supermacromolecular assemblies or multiple signaling interactions by 3, 4 or 5–way FRET in addition to the possibility of unmixing cellular autofluorescence from FRET data.

The data requirements and information gathered by N-Way FRET can be defined in terms of the number of fluorophores in the system. For N fluorophores, the number of images N_d_ (or samples on the excitation-emission landscape) scales as.
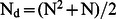



This is the number of peaks along the diagonal plus the upper half of the EEM (Fig. 1). It is important to note that the number of intermolecular interactions that can be measured scales faster than N. For two fluorophores only one interaction can be measured, three fluorophores can measure 3 interactions however, 4 fluorophores can measure 6 etc. Specifically, the number of interactions N_I_ that can be measured scales as:
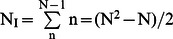



Together, these two constraints indicate that the number of fluorophores N increases, the data cost per interaction measured (N_d_/N_I_) will approach unity, yet the number of interactions will continue to grow as ∼N^2^. Thus, maximizing the number of fluorophores in the system will provide the greatest possible access to biochemical networks. Unfortunately, the visible spectrum is not large enough to accommodate more than about 5 or 6 fluorescent proteins. Nonetheless, approaching this limit will still afford significant gains provided that the practicalities of acquiring the data can be achieved. Specifically, 4-way FRET would require 10 images but could measure 6 interactions and 5-way FRET would require 15 images but could measure 10 interactions. While it is possible to measure 15 images on a single cell with subsecond kinetics, this requirement is compounded by the requirement that at least N-excitation wavelengths must be used in combination with N-emissions. Only a handful of commercial systems are currently capable of live-cell experiments for the 5-way FRET measurement, however this number is likely to grow as systems with spectral detection and multiple detectors are becoming more commonplace. Furthermore, as FP and molecular technologies improve, it may be possible to integrate N-Way FRET into high-content imaging systems to provide access to large-scale biochemical networks.

The selection of excitation and emission wavelengths should be chosen to optimize a balance between selectivity and signal-to-noise ratio (SNR) of the raw data. Previously, Neher and Neher [18] defined a figure of merit that could be used to determine this optimal balance for two-way FRET. Recent work on SNR in FRET measurements including Bayesian methods should help guide these choices [42]. Although additional work will be needed to extend these optimizations to N-Way FRET, the error propagation matrices defined here should provide a starting point.

N-Way FRET simplifies the mathematics of spectral FRET analysis, extends FRET methodology to account for three or more fluorophores and provides new error analysis tools for FRET microscopy. As a demonstration, N-Way FRET was able to quantify interactions between multiple Gag molecules but not the lipid raft marker, Fyn(10), in single living cells. Previously, this experiment required multiple experiments to draw this conclusion [17]. Future application of N-Way FRET to signal transduction studies will open new vistas by capturing the spatial and temporal organization of multiple signaling events within single cells. Additionally, application of N-Way FRET may also be used to improve quantitation in single-molecule FRET studies by treating the spectral matrices as probabilities for couplings between fluorescence spectra [43], [44]. This additional information should facilitate interpretation of changes in fluorescence signal amplitudes in terms of FRET efficiencies to improve distance estimates. Additionally, the use of these matrices may improve fluorescence lifetime imaging of FRET signals by facilitating the unmixing of overlapping fluorescence components [45].

We believe that the general formalism of N-Way FRET lays the foundation for approaching new FRET problems in wide range of applications, instruments and biological problems.

## Supporting Information

Figure S1 **Line diagram of the N-Way FRET microscope. A**) Schematic and **B**) list of the microscope’s components. Excitation light was provided by a fast switching 150 W xenon light source (Oligochrome, Till Photonics) using excitation bandpass filters (F1, F2 and F3). This light was reflected toward the sample by a custom polychromatic mirror (M1, Chroma) and focused through the objective lens (O1). Emitted fluorescence was reflected by M2 onto camera D2 after passing through associated relay optics and (L2 and L5) and emission bandpass filter (F5). Blue and red fluorescence were split by M4 and reflected onto cameras D1 and D3 after passing through emission bandpass filters (F4 and F6). **C)** Excitation and emission filter combinations used to generate the N-FRET spectral images.
(TIF)

Figure S2 **N-Way FRET accurately unmixes individual fluorophores.** CFP (**A**), YFP (**B**) or RFP (**C**) were expressed in COS7 cells and analyzed by N-Way FRET. Raw data displayed with each image autoscaled (top rows of **A, B** and **C**) showed that variable fluorophore fluorescence was observed in each image. In addition, cellular autofluorescence could be observed in some of the data (e.g. cc and rr images of **B** and cc, cy images of **C**). However, the unmixed N-Way FRET signals showed only concentrations of the expected fluorophore (bottom row, **A, B** and **C**). Quantification on a per cell basis (**D**, **E** and **F**) with signals were normalized to the peak unmixed fluorescence, confirmed that the expected fluorescence predominated. 20 cells per condition; error bars are standard deviation.
(TIF)

Figure S3 **N-Way FRET demonstrates insensitivity to non-interacting species.** COS7 cells expressing linked RFP-YFP construct in combination with free CFP were analyzed N-Way FRET. Raw data (A, top row) indicated fluorescence in all channels, however N-Way FRET analysis revealed that despite variations in total FP concentrations, FRET signals were only observed for the E_[YR]_ image as expected (A, bottom row). **B**) Quantification of these data (normalized to [R]) demonstrated that while the expression of CFP was higher and more variable than the linked construct and that the E_[YR]_ signal was clearly distinguishable and significantly different (p<0.05, by Tukey HSD post hoc comparison of means) above E_[CY]_ and E_[CR]_. 20 cells analyzed per condition; error bars are standard deviation.
(TIF)

Figure S4 **N-Way FRET error analysis of the FRET Stoichiometry ratios.** FRET Stoichiometry ratios for the apparent FRET efficiencies (E_A_ and E_D_) and molar ratios (R_M_) were computed for the energy transfer events C-Y (A, top row), Y-R (B, top row) and C-R (C, top row) from a cell expressing the YFP-CFP-RFP linked construct (same cell as Fig. 5). The propagated error images (coefficient of variation, CV, bottom row in each panel) showed that the periphery of the cell has the largest CV in the E_A_, E_D_ and R_M_ images. Importantly, the strength of the FRET efficiency of the E_A_^CY^ signal is about double E_A_^YR^ (compare **A** and **B** top rows), however their CVs are approximately equal (compare **A** and **B** bottom rows) illustrating that in addition to the FRET efficiency, shot noise associated with acquisition plays and significant role.
(TIF)

Figure S5 **Determination of FRET efficiencies by sequential acceptor photobleaching of the CFP-YFP-RFP construct. A**) The FRET efficiencies between the triple linked constructs were determined by capturing the intensity of CFP, YFP and RFP fluorescence from ROIs placed over cells cells expressing the CFP-YFP-RFP construct. Collimated laser light was used to bleach RFP (561 nm, frames 6–35) and then YFP (515 nm, frames 41–75). The FRET efficiencies were calculated as the increase in donor fluorescence, E = 1-F_AD_/F_D_, following each acceptor bleach. Some incidental photobleaching was observed for YFP during the acquisition, hence, the maximum intensity of YFP was taken for the FRET calculation during the RFP bleach. **B)** Quantification of data from multiple cells (error is standard deviation, n = 30 cells).
(TIF)

Figure S6
**Determination of FRET efficiencies of calibration constructs by fluorescence lifetime an acceptor photobleaching. A**) The FRET efficiencies were determined by acceptor photobleaching as in Fig. S5 for the tandem linked constructs of CFP-YFP, CFP-RFP and YFP-RFP (n = 8 cells each, error bars are the standard deviation). **B**) Determination of FRET efficiencies for the same three linked constructs as measured by fluorescence lifetime and computed as 
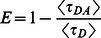
(n = 3–6 cells, propagated error). **C**) The average fluorescence lifetime (τAVE) and the lifetime weighted quantum yield <τ> = Σαiτi for the constructs used in computing B (n = 3–6 decays, error is standard deviation, χ2<1.3 for all regressions, 3-exponential model).(TIF)

Methods S1 **Supporting Methods.**
(DOCX)

Table S1 **Linked constructs used in this study.**
(DOCX)
