# Supplementary figures and images for: N-Way FRET Microscopy of Multiple Protein-Protein Interactions in Live Cells
Source: PLoS One. 2013 Jun 6;8(6):e64760. doi: 10.1371/journal.pone.0064760 (PMC3675202; doi:10.1371/journal.pone.0064760)

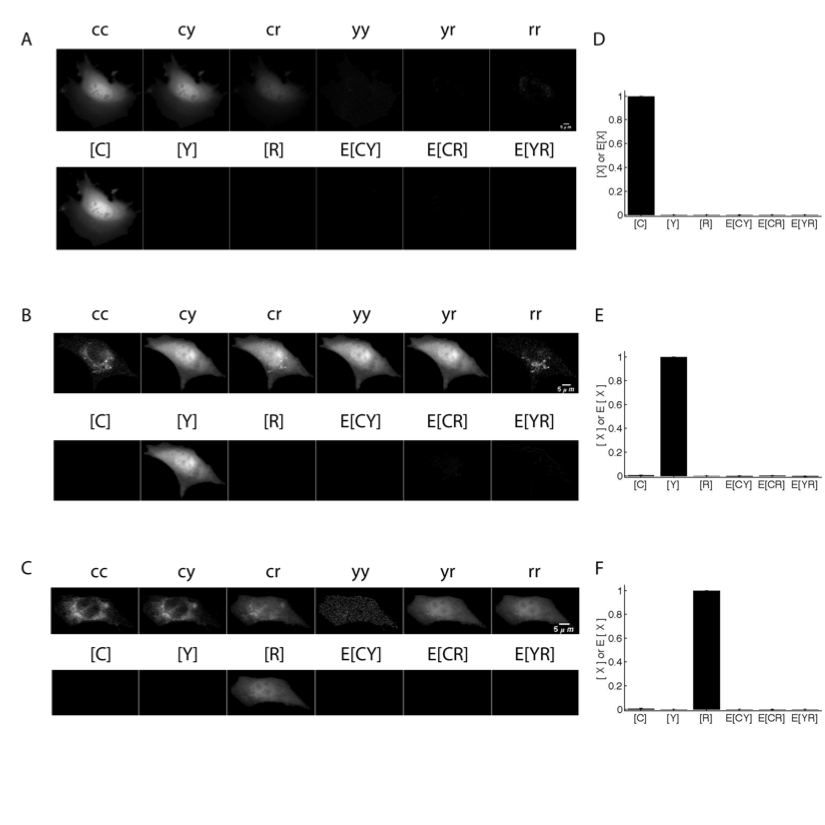

Supplement: Figure S2 — N-Way FRET accurately unmixes individual fluorophores. CFP (A), YFP (B) or RFP (C) were expressed in COS7 cells and analyzed by N-Way FRET. Raw data displayed with each image autoscaled (top rows of A, B and C) showed that variable fluorophore fluorescence was observed in each image. In addition, cellular autofluorescence could be observed in some of the data (e.g. cc and rr images of B and cc, cy images of C). However, the unmixed N-Way FRET signals showed only concentrations of the expected fluorophore (bottom row, A, B and C). Quantification on a per cell basis (D, E and F) with signals were normalized to the peak unmixed fluorescence, confirmed that the expected fluorescence predominated. 20 cells per condition; error bars are standard deviation. (TIF) [file pone.0064760.s002.tif]

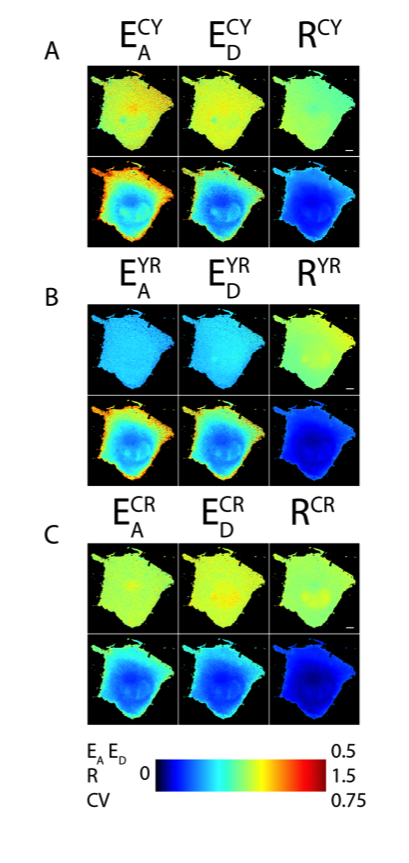

Supplement: Figure S4 — N-Way FRET error analysis of the FRET Stoichiometry ratios. FRET Stoichiometry ratios for the apparent FRET efficiencies (EA and ED) and molar ratios (RM) were computed for the energy transfer events C-Y (A, top row), Y-R (B, top row) and C-R (C, top row) from a cell expressing the YFP-CFP-RFP linked construct (same cell as Fig. 5). The propagated error images (coefficient of variation, CV, bottom row in each panel) showed that the periphery of the cell has the largest CV in the EA, ED and RM images. Importantly, the strength of the FRET efficiency of the EA CY signal is about double EA YR (compare A and B top rows), however their CVs are approximately equal (compare A and B bottom rows) illustrating that in addition to the FRET efficiency, shot noise associated with acquisition plays and significant role. (TIF) [file pone.0064760.s004.tif]

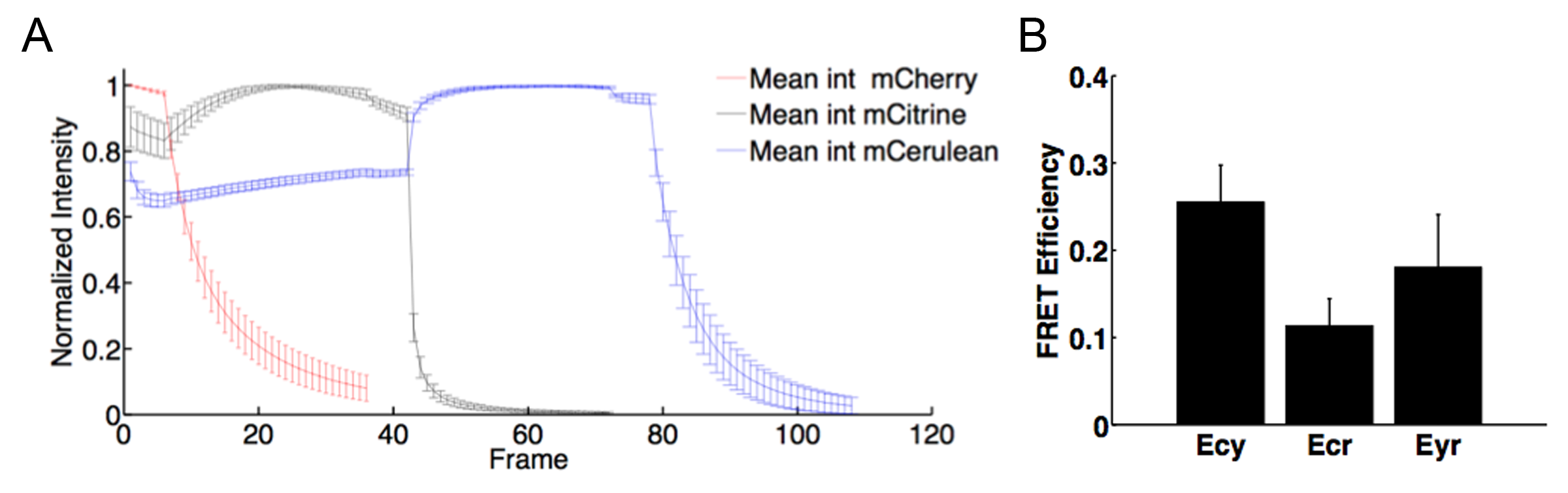

Supplement: Figure S5 — Determination of FRET efficiencies by sequential acceptor photobleaching of the CFP-YFP-RFP construct. A) The FRET efficiencies between the triple linked constructs were determined by capturing the intensity of CFP, YFP and RFP fluorescence from ROIs placed over cells cells expressing the CFP-YFP-RFP construct. Collimated laser light was used to bleach RFP (561 nm, frames 6–35) and then YFP (515 nm, frames 41–75). The FRET efficiencies were calculated as the increase in donor fluorescence, E = 1-FAD/FD, following each acceptor bleach. Some incidental photobleaching was observed for YFP during the acquisition, hence, the maximum intensity of YFP was taken for the FRET calculation during the RFP bleach. B) Quantification of data from multiple cells (error is standard deviation, n = 30 cells). (TIF) [file pone.0064760.s005.tif]

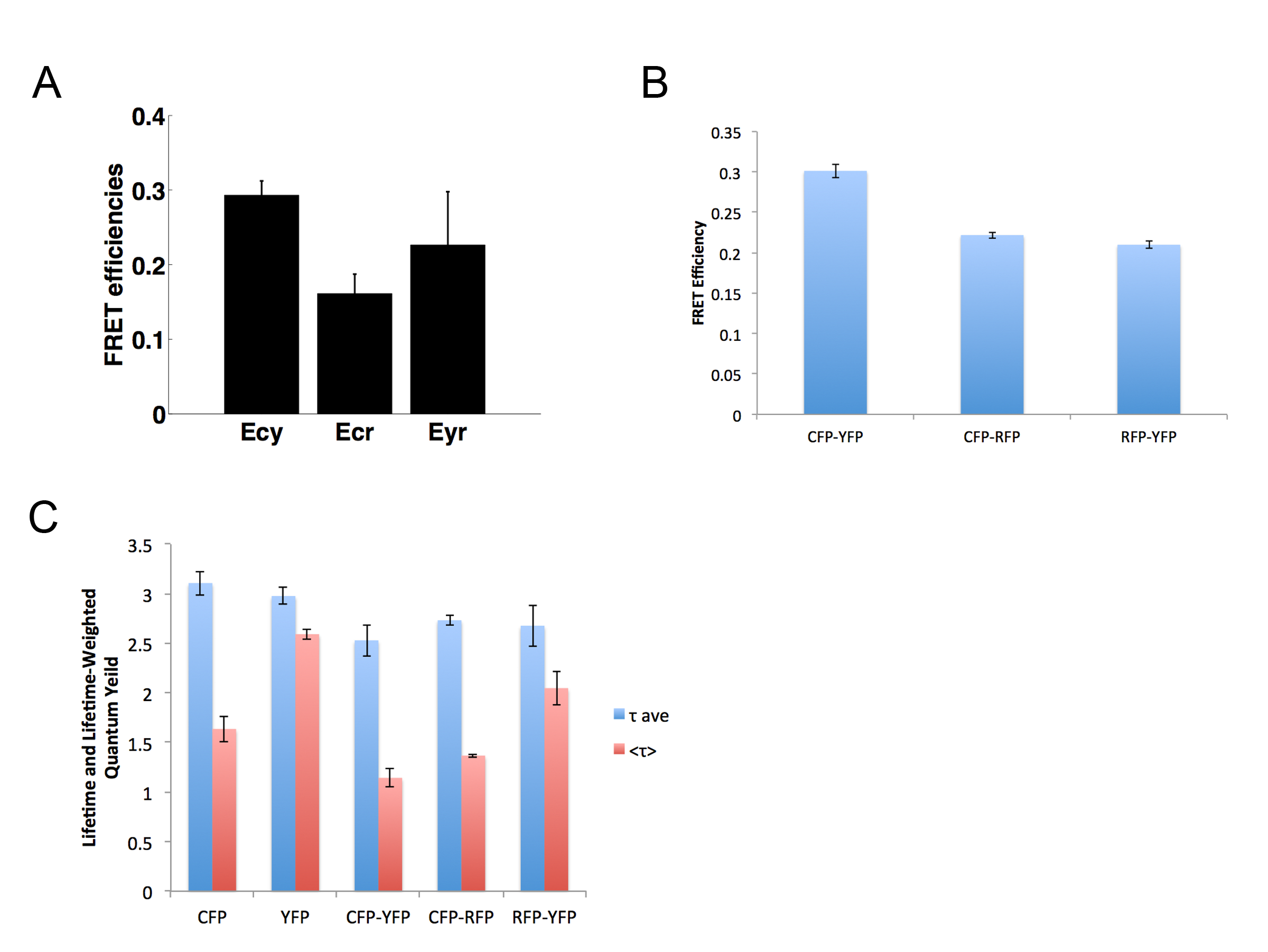

Supplement: Figure S6 — Determination of FRET efficiencies of calibration constructs by fluorescence lifetime an acceptor photobleaching. A) The FRET efficiencies were determined by acceptor photobleaching as in Fig. S5 for the tandem linked constructs of CFP-YFP, CFP-RFP and YFP-RFP (n = 8 cells each, error bars are the standard deviation). B) Determination of FRET efficiencies for the same three linked constructs as measured by fluorescence lifetime and computed as (n = 3–6 cells, propagated error). C) The average fluorescence lifetime (τAVE) and the lifetime weighted quantum yield <τ> = Σαiτi for the constructs used in computing B (n = 3–6 decays, error is standard deviation, χ2<1.3 for all regressions, 3-exponential model). (TIF) [file pone.0064760.s006.tif]
