# Supplementary material for: N-Way FRET Microscopy of Multiple Protein-Protein Interactions in Live Cells
Source: PLoS One. 2013 Jun 6;8(6):e64760. doi: 10.1371/journal.pone.0064760 (PMC3675202; doi:10.1371/journal.pone.0064760)
Supplement: Methods S1 — Supporting Methods. (DOCX) [file pone.0064760.s007.docx]

**Supporting Methods**

Plasmid Constructs Linked FP-FP and FP-FP-FP constructs were generated by PCR of fluorescent proteins with primers that added DNA sequences coding for amino acid linkers and restriction sites. These products were then cloned into the multiple cloning site of an acceptor plasmid containing one or two FPs and were based on the pEGFP-N1 or pEGFP-C1 Clontech backbone. The resulting constructs are shown in Table S1.

For measurement of a molecular assembly, we constructed fluorescent fusions to HIV-Gag and the lipid raft marker Fyn(10) by replacing the fluorescent protein in pCMV NL Gag-mCerulean with mCitrine and mCherry using overlap extension PCR cloning [1]. Briefly, oligos were used to amplify the reading frames of mCitrine and mCherry excluding the start and stop codons. The amplified products were purified and used as primers for amplification of the pCMV NL Gag-mCerulean vector. After digestion of the original vector with DpnI the amplified vectors were transformed in *E. coli*. Clones were screened by visualization of proper spectral properties after transfection in COS7 cells. Sequences were verified in clones displaying the proper spectral properties by DNA sequencing.

**Microscope Instrumentation**

Two microscopes were used in this study. Scope 1 was described previously [2]. Scope 2 was a custom-built iMIC (Till Photonics USA, Rochester, NY) designed to accommodate three emCCD cameras (iXon 885, Andor, Belfast, Northern Ireland) (Fig. 1S). This instrument used a 60x 1.2NA water immersion lens with correction collar (Olympus, Tokyo, Japan) to minimize spherical aberration of live samples. Cells were maintained at physiological temperature (37^o^C) in an in-house constructed heated enclosure on the top of the microscope. For all N-Way FRET experiments, images were captured by sequentially exciting the sample with c-excitation, y-excitation and r-excitation and simultaneously capturing images on all three cameras. Thus, for c-excitation, we obtain image combinations {cc, cy, cr}, for y-excitation {0, yy, yr} and r-excitation {0, 0, rr}. These images were then combined with zero images were removed into a single data vector **d** = {cc, cy, cr, yy, yr, rr}.

**References**

1. Bryksin, A. V., and I. Matsumura (2010) Overlap extension PCR cloning: a simple and reliable way to create recombinant plasmids. Biotechniques 48:463-465.

2. Hoppe AD, Shorte SL, Swanson JA, Heintzmann R (2008) 3D-FRET Reconstruction Microscopy for Analysis of Dynamic Molecular Interactions in Live Cells. pp. biophysj.107.125385.
