# Supplementary material for: N-Way FRET Microscopy of Multiple Protein-Protein Interactions in Live Cells
Source: PLoS One. 2013 Jun 6;8(6):e64760. doi: 10.1371/journal.pone.0064760 (PMC3675202; doi:10.1371/journal.pone.0064760)
Supplement: Table S1 — Linked constructs used in this study. (DOCX) [file pone.0064760.s008.docx]

Table S1. Linked constructs used in this study.

| **Construct** | **Amino Acid Linker** |
| --- | --- |
| C-Y | C-**SGLKDLELKLRILQSTVPRARDPPVAT**-Y (long) |
| R-C | R-**SGLKDLELKLRILQSTVPRARDPPVAT**-C (long) |
| R-Y | R-**SGLKDLELKLRILQSTVPRARDPPVAT**-Y (long) |
| R-dark(mCitrineY67C)-C | R-**SGLKDLE**-dark-**DPPVAT**-C |
| R-kinesin(Kif1a,1698aa)-C | R-**SGLKDLE**-kinesin-**KLRILQSTVPRARDPPVAT**-C |
| C-R-Y | C-**SGLKDLE**-R-**DPPVAT**-Y |
| R-C-Y | R-**SGLKDLE**-C-**DPPVAT**-Y |
